# High-Throughput Sorting of the Highest Producing Cell via a Transiently Protein-Anchored System

**DOI:** 10.1371/journal.pone.0102569

**Published:** 2014-07-18

**Authors:** Kuo-Hsiang Chuang, Yuan-Chin Hsieh, I-Shiuan Chiang, Chih-Hung Chuang, Chien-Han Kao, Ta-Chun Cheng, Yeng-Tseng Wang, Wen-Wei Lin, Bing-Mae Chen, Steve R. Roffler, Ming-Yii Huang, Tian-Lu Cheng

**Affiliations:** 1 Graduate Institute of Pharmacognosy, Taipei Medical University, Taipei, Taiwan; 2 Graduate Institute of Medicine, Kaohsiung Medical University, Kaohsiung, Taiwan; 3 Department of Biomedical Science and Environmental Biology, Kaohsiung Medical University, Kaohsiung, Taiwan; 4 Department of Biochemistry, College of Medicine, Kaohsiung Medical University, Kaohsiung, Taiwan; 5 Institute of Biomedical Sciences, National Sun Yat-Sen University, Kaohsiung, Taiwan; 6 Institute of Biomedical Sciences, Academia Sinica, Taipei, Taiwan; 7 Department of Radiation Oncology, Cancer Center, Kaohsiung Medical University Hospital, Kaohsiung Medical University, Kaohsiung, Taiwan; 8 Cancer Center, Kaohsiung Medical University Hospital, Kaohsiung, Taiwan; 9 Ph.D. Program for Clinical Drug Discovery from Botanical Herbs, Taipei Medical University, Taipei, Taiwan; 10 Master Program for Clinical Pharmacogenomics and Pharmacoproteomics, Taipei Medical University, Taipei, Taiwan; Imperial College London, United Kingdom

## Abstract

Developing a high-throughput method for the effecient selection of the highest producing cell is very important for the production of recombinant protein drugs. Here, we developed a novel transiently protein-anchored system coupled with fluorescence activated cell sorting (FACS) for the efficient selection of the highest producing cell. A furin cleavage peptide (RAKR) was used to join a human anti-epithelial growth factor antibody (αEGFR Ab) and the extracellular-transmembrane-cytosolic domains of the mouse B7-1 antigen (B7). The furin inhibitor can transiently switch secreted αEGFR Ab into a membrane-anchored form. After cell sorting, the level of membrane αEGFR Ab-RAKR-B7 is proportional to the amount of secreted αEGFR Ab in the medium. We further selected 23 αEGFR Ab expressing cells and demonstrated a high correlation (R^2^ = 0.9165) between the secretion level and surface expression levels of αEGFR Ab. These results suggested that the novel transiently protein-anchored system can easily and efficiently select the highest producing cells, reducing the cost for the production of biopharmaceuticals.

## Introduction

Using mammalian cell systems to produce recombinant protein drugs has become a mainstream practice in biopharmacy. Owing to the post-translational modification and glycosylation patterns of proteins, such systems often cannot be effectively replaced by other systems, whether bacterial, yeast, plant, or insect cell systems, such that more than 50% of the therapeutic proteins on the market are produced by mammalian cell systems [1], [2]. Determining the most effective method for screening the highest producing mammalian cells is one of the greatest challenges in the protein drug development process.

Limiting dilution cloning (LDC) is the most commonly used method due to its relative simplicity and low cost [3]. However, the whole process is time-consuming and labor-intensive, and only a few hundred clones can be certainly characterized, increasing the chance to lose highest producing cells. To overcome this problem, the fluorescence activated cell sorter (FACS) which can accurately analyze and separate single cells or specific subpopulations in short time has been increasingly used to identify high producing cells in the biopharmaceutical industries [4], [5]. Nevertheless, secreted proteins can usually not stay on cell surface, resulting in the difficult of measurement on single cells. Recently, researchers have developed different selection methods based on the co-expression of a non-fluorescent surface molecule (ex: CD20) [6] or a fluorescent intracellular protein (ex: GFP) [7] by inducing additional internal ribosome entry sites (IRESs) for reporter protein expression [8]. Some drawbacks, however, such as the possible cytotoxicity of fluorescent proteins [9], the limitation of cell line specific characteristics [10], and lower expression levels of downstream reporter proteins in the IRES system [11], affect the accuracy of the selection of high-producing cells. Other methods which immobilize secreted proteins on a cell, including matrix-based secretion assay [12], gel micro drop technology [13], [14], and GPI-anchored systems [15], require skillful laboratory personnel and expensive instruments, which may prevent their routine use [12]–[14], [16]. In short, a strategy that is easy to operate, low in cost, and FACS compatible is still unavailable for high protein-producing cell selection.

**Figure 1:**
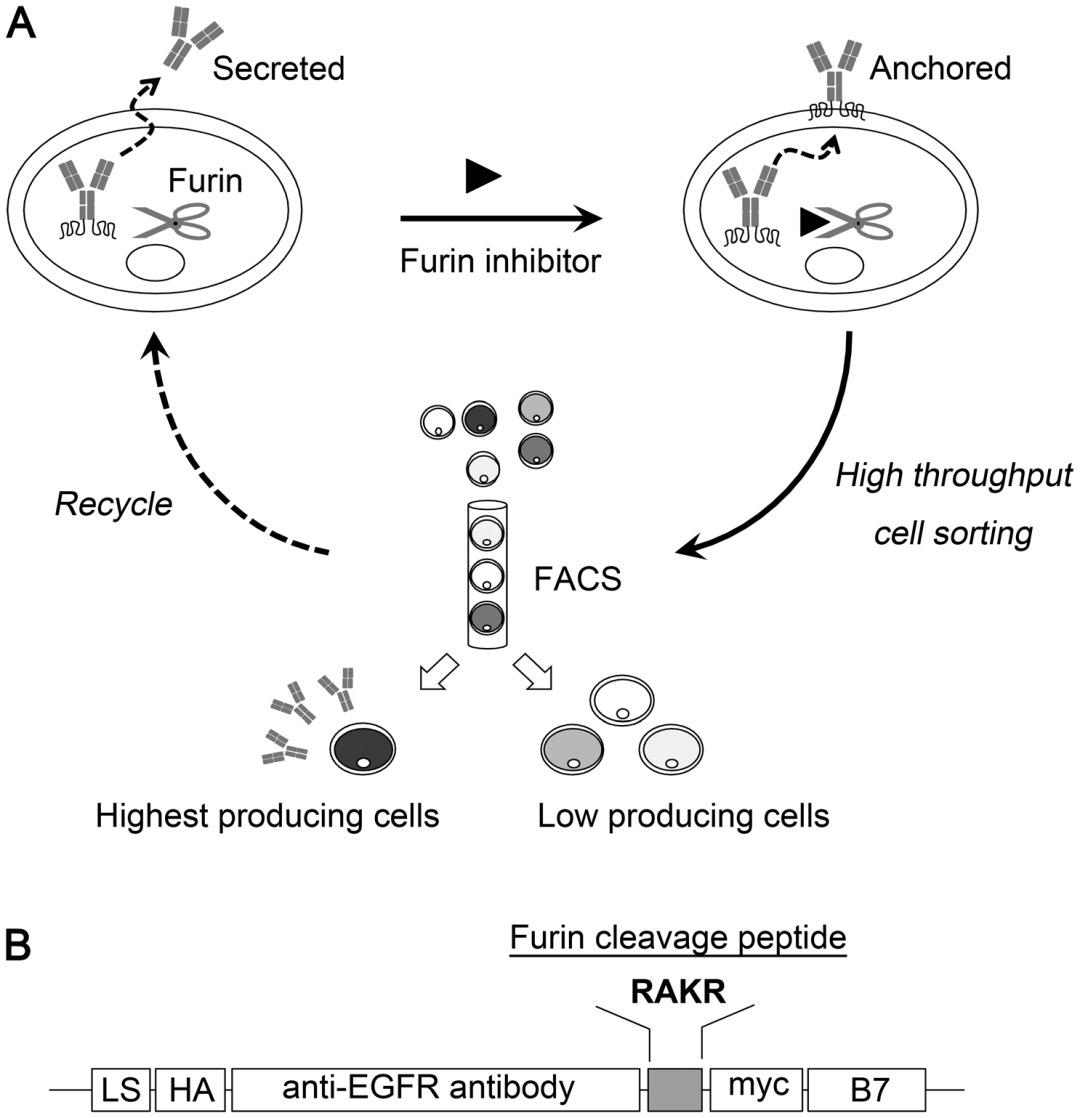
High-throughput sorting of the highest protein-productive cell by a transiently protein-anchored system. (A) Strategy and organization of the transiently protein-anchored system. (B) Schematic representation of the gene construction of the transiently protein-anchored system using anti-EGFR antibody as an example. The construction includes, from N to C termini, an immunoglobulin leader sequence (LS), an HA epitope, the anti-EGFR antibody fragment, the furin cleavage site (RAKR), and the immunoglobulin C2-type extracellular-transmembrane-cytosolic domains of murin B7-1 antigen (B7).

In this study, we developed a novel transiently protein-anchored system coupled with FACS for efficient selection of the highest protein secreting cells. A furin cleavage peptide (RAKR) was used as a linker between a secreted αEGFR Ab and the extracellular-transmembrane-cytosolic domain of mouse B7-1 antigen (B7). The furin protease in the Golgi apparatus can efficiently cut the RAKR peptide to allow the αEGFR Ab to be secreted. Moreover, in the presence of furin inhibitor the secreted αEGFR Ab can be switched to a membrane-anchored αEGFR Ab-RAKR-B7 protein for screening the highest producing cell by FACS (Figure 1). First, RAKR fused secretory protein was confirmed to be released after the digestion by furin protease in the Golgi apparatus in HEK-293. Then, the switch of the secreted αEGFR Ab to an anchored form was examined in the presence of the furin inhibitor Dec-RVKR-CMK by using flow cytometry, ELISA, and western blot. Finally, we further selected 23 clones of αEGFR Ab expressing cells and calculated the correlation between the amounts of secreted αEGFR Ab and the membrane-anchored αEGFR Ab-RAKR-B7 levels. Positive results indicated that our system is a high-throughput method for the selection of the highest producing cells to meet the needs of biopharmaceutical markets.

## Materials and Methods

### Reagents and cells

Furin convertase inhibitor Decanoyl-Arg-Val-Lys-Arg-chloromethyl–ketone (Dec-RVKR-CMK) was purchased from Enzo Life Sciences, Inc. Human embryonic kidney 293 cells (HEK-293) were purchased from American Type Culture Collection (ATCC). The cells were cultured in Dulbecco minimal essential medium (Sigma–Aldrich) supplemented with 10% heat-inactivated bovine calf serum, penicillin (100 units/mL), and streptomycin (100 mg/mL) at 37°C in a humidified atmosphere of 5% CO2. Phoenix cells were purchased from Orbigen. The cells were cultured in Dulbecco minimal essential medium F12 (Sigma–Aldrich) supplemented with 10% heat-inactivated fetal bovine serum, penicillin (100 units/mL), and streptomycin (100 mg/mL) at 37°C in a humidified atmosphere of 5% CO2.

### Plasmid construction

The extracellular-transmembrane domain derived from mouse B7-1 antigen (B7) was described in a previous study [17], [18]. We created a furin cleavage site (RAKR) as a linker in the N-terminus of the extracellular-transmembrane-cytosolic domain of B7 by polymerase chain reaction. The complementary DNA sequence encoding furin cleavage site (RAKR sequence) and the extracellular-transmembrane domain of B7 were then constructed in pLKO-As3w-puro lentiviral vector.

The humanized anti-EGFR antibody h528 was described in the research of K. Makabe et al [19]. The V_L_ and V_H_ segments of humanized anti-EGFR antibody were synthesized by assembly polymerase chain reaction [20]. The complete light chain/heavy chain of the antibody was then established by fusion of the V_L_/V_H_ segments with their constant region. The constant regions, including the C_κ_ and C_H_1-hinge-C_H_2-C_H_3 domain, were cloned from complementary DNA prepared from Raji cells. The heavy chain and the light chain were joined by a FMDV 2A self-processing peptide [21] in the pLKO-As3w-puro lentiviral vector to generate pLKO-As3w-αEGFR Ab-RAKR-B7.

### Generation of αEGFR Ab-secreting cell by lentiviral transduction

To produce pseudotyped lentiviruses, pLKO-As3w-αEGFR Ab-RAKR-B7 was co-transfected with pMD.G and pCMVΔR8.91 to Phoenix cells (Orbigen) by TransIT-LT1 (Mirus). After transfection, the culture medium was filtered and collected in 24, 48, and 72 hours. Ten-fold concentrated medium containing lentivirus was mixed with 8 µg/ml polybrene (sigma-Aldrich), and that mixture was added to HEK-293 cells. Following lentiviral transduction, cells were selected in puromycin-containing medium (1 µg/ml) to generate HEK-293/αEGFR cells that secreted αEGFR Ab in standard culture condition.

### Western blotting analysis

HEK-293/αEGFR cells were cultured in serum-free DMEM medium containing furin inhibitor (0, 2, and 50 µM). After 24 hours, cultured medium and cells (5×10^4^) were collected and heated in reducing sample buffer at 100°C for 10 minutes. The samples were electrophoresed in an 8% SDS polyacrylamide gel under reducing conditions and then transferred onto a nitrocellulose paper (Millipore). After blocking in 5% skim milk, the membrane was incubated with 1 µg/mL horseradish peroxidase-conjugated goat anti-human IgG Fc antibody (Jackson ImmunoResearch Laboratories). The blots were washed three times with PBS-T (PBS containing 0.05% Tween 20) and twice with PBS before specific bands were visualized by enhanced chemiluminescence detection according to the manufacturer's instructions (Pierce).

### Flow cytometry analysis

HEK-293 and HEK-293/αEGFR cells were cultured in serum-free DMEM medium containing furin inhibitor (20 µM). After 24 hours, cells were collected and washed by PBS. The expression level of anchored-αEGFR Ab-RAKR-B7 proteins was measured by staining cells with a FITC-conjugated goat anti-human IgGFcγ antibody (Jackson ImmunoResearch Laboratories) in PBS containing 0.05% bovine serum albumin on ice. After removing unbound antibodies by extensive washing in PBS, the surface immunofluorescence of viable cells was measured with a flow cytometer (FACScalibur, BD Biosciences, Mountain View, CA) and fluorescence intensities were analyzed with CellQuest (BD Biosciences).

### ELISA analysis

HEK-293 and HEK-293/αEGFR cells were seeded (1.5×10^6^ per well) overnight in 6-well plates. And then the cultured medium was shifted into serum-free DMEM medium or serum-free DMEM medium containing furin inhibitor (20 µM). The cultured medium was added to Maxisorp 96-well microplates (50 µL/well) for 1 hour at 37°C. Plates were then blocked with 5% skim milk in PBS for 1 hour at 37°C. After being washed, the wells were incubated with horseradish peroxidase (HRP)-conjugated anti-human IgG Fc antibody (Jackson ImmunoResearch Laboratories). The plates were washed three times with PBS-T and twice with PBS, and bound peroxidase was measured by adding 150 µL/well ABTS solution [0.4 mg/mL, 2,2′-azinobis(3-ethylbenzthiazoline -6-sulfonic acid) (ABTS; Sigma- Aldrich), 0.003% H2O2, and 100 mM phosphate-citrate, pH = 4.0] for 30 min at room temperature. Color development was measured at 405 nm on a microplate reader. All the readings were background-adjusted by subtracting the absorbance of a blank control in the ELISA procedures.

## Results

### Furin inhibitor-mediated switch of the secreted αEGFR Ab into anchored form

The coding sequence of αEGFR Ab is joined to the mouse C2-type extracellular-transmembrane-cytosolic domains of the B7-1 receptor (B7) through a furin cleavage site (RAKR) to form αEGFR Ab-RAKR-B7 in a lentiviral vector. The HEK-293 cells were infected with lentiviral particles and selected with puromycin to obtain HEK-293/αEGFR cells. The furin protease in the Golgi apparatus can efficiently cut the RAKR linker in HEK-293/αEGFR cells, allowing αEGFR Ab secretion. Only 2 amino acid residues (RA) of the furin cleavage site were left at the C-terminal end of the αEGFR Ab heavy chain after Golgi processing (Figure 1B).

**Figure 2:**
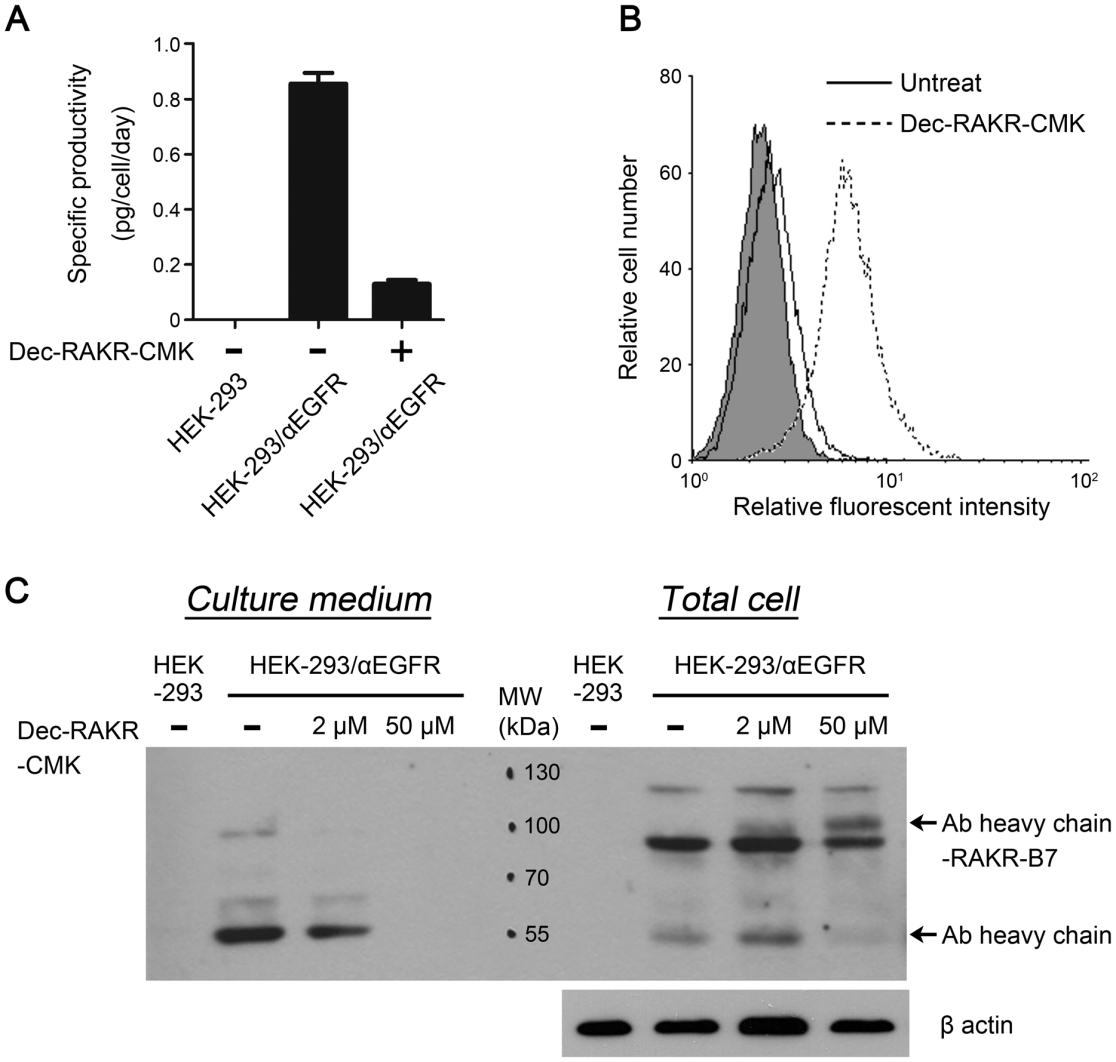
Furin inhibitor-mediated switch of secreted αEGFR Ab and anchored αEGFR Ab-RAKR-B7. (A) HEK-293/αEGFR cells were treated with 20 µM furin inhibitor for 24 h, and HEK-293 cells were used as a negative control. Cultured medium was coated on 96-well plates and were detected by ELISA using HRP conjugated goat anti-human IgGFcγ antibody. Bars, SD. (B) HEK-293 cells (filled histogram); HEK-293/αEGFR cells (solid line) and HEK-293/αEGFR cells treated with furin inhibitor (20 µM) (dashed line) were analyzed by flow cytometry using a specific antibody to the human IgGFcγ to assess the expression of membrane-anchored αEGFR Ab-RAKR-B7. (C) The cultured medium and cell lysate harvested from HEK-293 cells, HEK-293/αEGFR cells, and HEK-293/αEGFR cells treated with furin inhibitor (2 or 50 µM) were analyzed by western blotting using HRP conjugated goat anti-human IgGFcγ antibody. The clear bands appearing at 55 KD in lanes 2 and 3 correspond to the secreted αEGFR Ab heavy chain, and the clear bands appearing at 95 KD in lanes 7 and 8 correspond to the membrane-anchored αEGFR Ab heavy chain-RAKR-B7. The bands appearing at 80 KD in lanes 7, 8, and 9 correspond to the αEGFR Ab light chain-2A-heavy chain, and the bands appearing at 120 KD in lanes 7, 8, and 9 correspond to the αEGFR Ab light chain-2A-heavy chain-RAKR-B7.

To investigate whether the addition of the furin inhibitor (Dec-RVKR-CMK) [22] can switch the expression of the αEGFR Ab from secreted form to membrane form, HEK-293/αEGFR cells were treated with the furin inhibitor for 24 hours. The culture medium was collected and analyzed to determine the concentration of secreted αEGFR Ab by ELISA, while the surface level of αEGFR Ab-RAKR-B7 on the HEK-293 cells was analyzed simultaneously by flow cytometry. Figure 2A shows that HEK-293/αEGFR cells can continually secrete αEGFR Ab into cultured medium (0.85 pg/cell/day). After treatment of 20 uM furin inhibitor, the secretory level of αEGFR antibody was dramatically reduced (0.17 pg/cell/day). Similarly, Figure 2B shows that furin inhibitor treated HEK-293/αEGFR cells exhibited higher fluorescent intensity than the untreated cells. These results indicated that the furin inhibitor can efficiently modulate the switch of αEGFR Ab from secreted form to membrane-anchored αEGFR Ab-RAKR-B7 form in HEK-293/αEGFR cells. To confirm the furin inhibitor-mediated uncleavage of αEGFR Ab-RAKR-B7 and the reduction of the secreted αEGFR antibody, the cultured medium and cell lysate of HEK-293/αEGFR cells with or without furin inhibitor were separated by SDS-PAGE under reducing condition and analyzed by western blotting using human Fc domain specific antibody. Figure 2C shows that most of the αEGFR antibodies in the culture medium were in the secreted form with apparent molecular weights of approximately 55 kDa and that the amount of antibody secretion was decreased with increasing furin inhibitor. In contrast, the expression level of αEGFR Ab-RAKR-B7 fusion proteins (95 kDa) in cell lysate was increased when the concentration of the furin inhibitor was increased. These results indicated that the furin inhibitor can successfully modulate the switch of αEGFR Ab from the secreted form to the anchored form by preventing the furin-mediated cleavage of RAKR substrate peptide.

### Good correction between the secreted and the membrane-anchored αEGFR Ab

**Figure 3:**
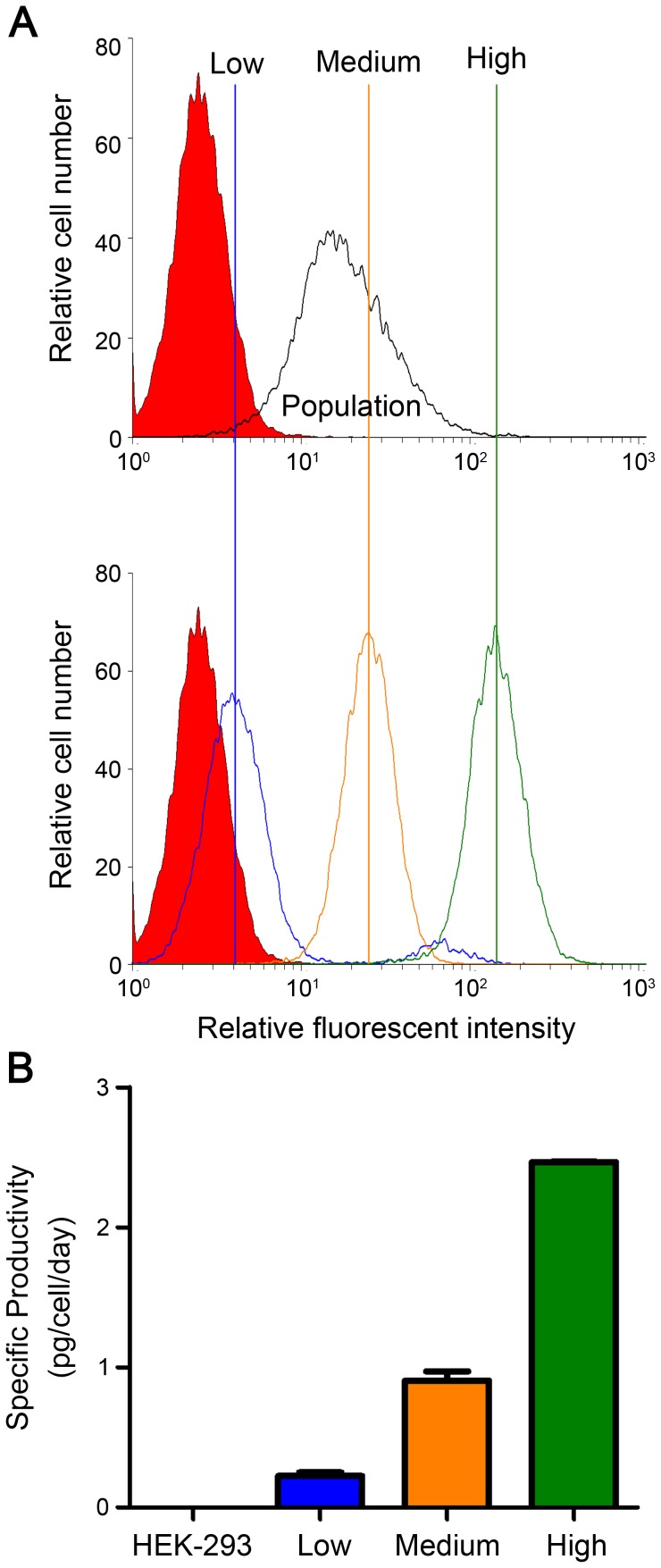
Correlation between the secreted αEGFR Ab and the membrane-anchored αEGFR Ab-RAKR-B7. (A) The HEK-293/αEGFR cells with high (green), medium (orange), or low (blue) expression levels of anchored αEGFR Ab-RAKR-B7 were sorted by flow cytometry. Original populations or sorted clones of HEK-293/αEGFR cells were confirmed by first being treated with furin inhibitor (20 µM), and then through analysis by flow cytometry using FITC conjugated goat anti-human IgGFcγ antibody. (B) The cultured media of sorted HEK-293/αEGFR cells were collected and analyzed by ELISA using HRP conjugated anti-human IgGFcγ antibody. Bars, SD.

To assess whether the expression level of membrane αEGFR Ab-RAKR-B7 can reflect the amount of secreted αEGFR Ab, HEK-293/αEGFR cells were treated with furin inhibitor, and were sorted into three populations according to the high, medium, or low expression levels of membrane αEGFR Ab-RAKR-B7 detected by flow cytometry (Figure 3A). After the removal of the furin inhibitor, the amount of αEGFR Ab in the cultured medium was measured by ELISA. Figure 3B shows that the HEK-293/αEGFR cells with high, medium, or low membrane αEGFR Ab-RAKR-B7 levels secreted 2.46, 0.91, and 0.22 pg/cell/day αEGFR Ab into the medium, respectively. These results indicated that the expression level of membrane αEGFR Ab-RAKR-B7 on the HEK-293/αEGFR cell is proportional to the amount of secreted αEGFR Ab in the medium.

### The correlation between the secreted and the anchored αEGFR Ab of 23 selected clones

**Figure 4:**
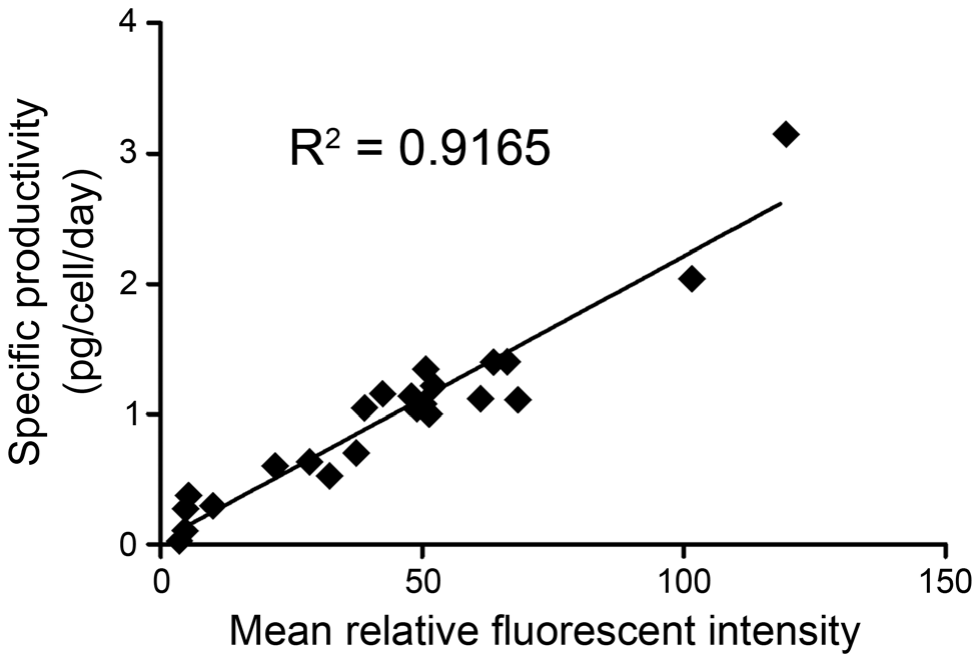
Calculation of the correlation coefficient between the secreted αEGFR Ab and the membrane-anchored αEGFR Ab-RAKR-B7. Each dot represents a monoclonal cell isolated from HEK-293/αEGFR cells. The cultured medium was collected from each clone and the secretion level of αEGFR Ab was determined by ELISA; the monoclonal cells were treated with furin inhibitor (20 µM) and analyzed by flow cytometry. The Y-axis represents the titer of secreted αEGFR Ab in the absence of furin inhibitor. The X-axis represents the mean relative fluorescent intensity of membrane-anchored αEGFR Ab-RAKR-B7 stained with FITC conjugated anti-human IgGFcγ antibody. Collectively, the titer of secreted αEGFR Ab and the surface expression level of anchored αEGFR Ab-RAKR-B7 reveal a highly positive correlation (R^2^ = 0.9165).

To further investigate the correlation of antibody titers between the secreted and the membrane-anchored αEGFR cells, HEK-293/αEGFR cells were sorted into 96-well plates at a density of one cell per well. Twenty-three HEK-293/αEGFR clones with various expression levels of membrane αEGFR Ab-RAKR-B7 were verified in the presence of furin inhibitors, and then selected for further analysis. The titers of secreted αEGFR Ab from these clones were measured by ELISA and plotted against the fluorescence intensity of respective membrane αEGFR Ab-RAKR-B7 in the presence of furin inhibitor. As shown in Figure 4, there was a significant correlation, with a correlation coefficient of 0.9165, between the titer of secreted αEGFR Ab and the fluorescent intensity of membrane αEGFR Ab-RAKR-B7. Thus, the expression level of membrane αEGFR Ab-RAKR-B7 is thought to be representative of the productivity of secreted αEGFR Ab. These results suggested that the transiently protein-anchored system can be easily and efficiently used to select the highest protein-producing cells.

## Discussion

Here, we describe a novel transiently protein-anchored system for efficient isolation of high producing mammalian cells. In the presence of furin inhibitor Dec-RVKR-CMK, the secreted αEGFR Ab can be transiently converted into the membrane-anchored αEGFR Ab-RAKR-B7. Importantly, the level of secreted αEGFR Ab is strongly correlated with the level of the membrane-anchored αEGFR Ab-RAKR-B7, allowing us to accurately isolate the most productive clones. The strategy will provide an effective tool for screening the highest protein-producing cell in a cost-effective and high throughput manner. Adapting this system to select the highest producing clone of cGMP banked CHO cells which stably secrete recombinant protein drugs will meet the requirements of the biopharmaceutical industry.

The tethering efficacy of the transmembrane domain-fused antibody was a critical concern in our screening system. Based on our previous studies, the optimized B7-1 membrane-tethered domain consists of a partial extracellular domain (143–245 a.a.), a transmembrane domain (246–267 a.a.), and an entire cytoplasmic tail (268–306 a.a.) [17], [18]. Two noteworthy issues of our construct were described as follows: First, the intact cytoplasmic tail is able to facilitate surface transportation of fusion proteins. Similar to previous reports, sorting elements of the cytoplasmic domain have indicated an ability to assist fused proteins for their translocation of proteins [23]. In addition, due to a lack of ER/Golgi retention signals, the cytoplasmic tail was revealed to lengthen the half-life of the tethered-proteins on the cell membrane [23]. Second, similar to many endogenous receptors that contained O-linked glycosylation residues, five glycosylations were observed in the extracellular domain (143–245 a.a.) of B7-1. Surface receptors has been rapid proteolyed once these O-linked glycosylations were removed. Thus, the retension of the extracellular domain was believed to be glycosylated and to able to stabilize the surface expression of fused proteins. Taken together, the antibody was fused to the optimalized membrane-tethered domain contained a glycosylated residue in the outer (exoplasmic) leaflet and an entire cytoplasmic sequence in the inner (cytoplasmic) leaflet. By using FACS, our fused antibody was confirmed to be stably expressed on the cell surface.

Although surface tethering is critical in high-throughput screening, massive and continual secretion of the target protein is the highest priority issue for industrial application. The furin cleavage sequence (-RAKR-) was used as a linker between the recombinant protein and the B7 membrane-tethered domain to meet both demands. Generally, furin localized in trans-Golgi network (TGN) is known to be involved in protein maturation [24]–[26]. Since all synthesized proteins were processed and sorted via TGN, recombinant proteins containing RAKR sequence should have been separated from the B7-1 tethering protein and consequently released into extracellular space. Based on previous studies, the furin cleavage sequence (-RAKR-) has also been applied as a linker in dual protein expression system. In their construct, two independent proteins, separated by a RAKR sequence, were coded from a single open reading frame and were capable of being effectively expressed. Similarly, a furin cleavage sequence (-RAKR-) has also been utilized in prevailing antibody-expression vectors in industrial production [27]. Our transiently protein-anchored system also utilizes a RAKR sequence, combining it with a furin inhibitor for efficient selection of high protein or antibody producing cell lines. This will improve the efficiency of protein drug manufacturing for the global protein therapeutics market.

The transiently protein-anchored system can also be applied to mammalian cell display systems as an improved platform for protein evolution and for library screening. By constructing a transmembrane domain to the target protein that was derived from a cDNA library, several studies have exhibited high-throughput screening of the protein of interest on cell membranes by FACS [15], [28], [29]. For example, Ho and colleagues constructed an anti-CD22 scFv-PDGFR transmembrane domain combinatory library with a randomizing intrinsic antibody hot spot and then transfected the library into HEK-293T cells, allowing them to achieve high-throughput screening of a mutant clone with increasing binding affinity for CD22 [30]. However, the selected clones need extra cloning to obtain the free-form of the target protein, which may limit the sample size for further analysis. For instance, ELISA and Biacore analyses were required to determine the binding affinity of antibodies [31], [32]; thus, the sketchy assessment or the small sample size may result in the loss of potential candidates. Compared to these systems, our transiently protein-anchored system makes it almost effortless to obtain the free-form of the target protein and offer a comprehensive platform for library screening.

## Conclusions

The transiently protein-anchored system possesses several advantages, including: (1) The system makes it easy to modulate the construction of the recombinant protein (released or membrane-bounded) by using furin inhibitors; (2) The system can be combined with FACS to effectively collect cells with the highest level of target proteins; (3) Without complicated processes or procedures to immobilize secreted protein on a cell, the system enhances the speed of clone selection and effectively reduces the costs of meeting the requirements of biopharmaceutical markets; (4) The surface expression level of anchored proteins is highly proportional to the secretion level of proteins, providing an accurate indicator for clone selection; (5) This system can also be applied to mammalian cell display systems to improve the development of protein drugs, including for affinity maturation and the selection of antibodies, as well as for the revolution or activity improvement of enzymes. Overall, we believe that the transiently protein-anchored system should provide an easy-to-use, cost-effective, and high throughput tool for the production of recombinant proteins, and could accelerate the process required for protein drugs to enter clinical pharmaceutical markets.
